# Supplementary material for: Modeling glioblastoma heterogeneity as a dynamic network of cell states
Source: Mol Syst Biol. 2021 Sep 16;17(9):e10105. doi: 10.15252/msb.202010105 (PMC8444284; doi:10.15252/msb.202010105)
Supplement: Supplementary file 6 — Source Data for Figure 5 [file MSB-17-e10105-s004.zip › Figure5A_sourcedata/GSEA_3017/nosigdb_stateA.GseaPreranked.1622795026485/RICHARDS_NOWAKOWSKI_SCIENCE_2017_VRG_UPREG.html]

Details for gene set RICHARDS\_NOWAKOWSKI\_SCIENCE\_2017\_VRG\_UPREG[GSEA]

|  || Dataset | state53017 |
| Phenotype | NoPhenotypeAvailable |
| Upregulated in class | na\_neg |
| GeneSet | RICHARDS\_NOWAKOWSKI\_SCIENCE\_2017\_VRG\_UPREG |
| Enrichment Score (ES) | -0.40640813 |
| Normalized Enrichment Score (NES) | -2.5691392 |
| Nominal p-value | 0.0 |
| FDR q-value | 6.2499996E-5 |
| FWER p-Value | 0.001 |
Table: GSEA Results Summary

  

Fig 1: Enrichment plot: RICHARDS\_NOWAKOWSKI\_SCIENCE\_2017\_VRG\_UPREG      
 Profile of the Running ES Score & Positions of GeneSet Members on the Rank Ordered List

  

| PROBE | GENE SYMBOL | GENE\_TITLE | RANK IN GENE LIST | RANK METRIC SCORE | RUNNING ES | CORE ENRICHMENT || 1 | FABP5 |  |  | 0 | 1.759 | 0.0354 | No |
| 2 | DBI |  |  | 17 | 0.683 | 0.0311 | No |
| 3 | PPA1 |  |  | 19 | 0.668 | 0.0434 | No |
| 4 | CA12 |  |  | 24 | 0.645 | 0.0518 | No |
| 5 | VIM |  |  | 46 | 0.543 | 0.0390 | No |
| 6 | FGFBP3 |  |  | 75 | 0.477 | 0.0169 | No |
| 7 | TGIF1 |  |  | 107 | 0.447 | -0.0092 | No |
| 8 | ZEB1 |  |  | 109 | 0.446 | -0.0014 | No |
| 9 | CTBP2 |  |  | 142 | 0.413 | -0.0293 | No |
| 10 | DNAJC1 |  |  | 151 | 0.407 | -0.0302 | No |
| 11 | TNC |  |  | 159 | 0.402 | -0.0300 | No |
| 12 | BCAN |  |  | 276 | 0.334 | -0.1546 | No |
| 13 | SEPT9 |  |  | 284 | 0.331 | -0.1559 | No |
| 14 | CACYBP |  |  | 288 | 0.330 | -0.1526 | No |
| 15 | MLLT10 |  |  | 311 | 0.322 | -0.1711 | No |
| 16 | HSPB1 |  |  | 312 | 0.322 | -0.1646 | No |
| 17 | IDI1 |  |  | 334 | 0.314 | -0.1820 | No |
| 18 | CREB5 |  |  | 407 | 0.291 | -0.2577 | No |
| 19 | NFIA |  |  | 413 | 0.289 | -0.2576 | No |
| 20 | DOCK1 |  |  | 414 | 0.289 | -0.2517 | No |
| 21 | SHC3 |  |  | 421 | 0.288 | -0.2527 | No |
| 22 | ACBD5 |  |  | 422 | 0.288 | -0.2469 | No |
| 23 | SLITRK2 |  |  | 458 | 0.279 | -0.2809 | No |
| 24 | MT2A |  |  | 473 | 0.275 | -0.2913 | No |
| 25 | PSAT1 |  |  | 491 | 0.271 | -0.3051 | No |
| 26 | REEP3 |  |  | 496 | 0.271 | -0.3041 | No |
| 27 | ZFP36L1 |  |  | 532 | 0.264 | -0.3384 | No |
| 28 | ITGA6 |  |  | 544 | 0.261 | -0.3456 | No |
| 29 | SOX9 |  |  | 577 | 0.254 | -0.3768 | No |
| 30 | ITGAV |  |  | 599 | -0.250 | -0.3955 | No |
| 31 | NES |  |  | 603 | -0.252 | -0.3938 | No |
| 32 | CD99 |  |  | 604 | -0.252 | -0.3887 | No |
| 33 | ABAT |  |  | 617 | -0.259 | -0.3971 | No |
| 34 | RGS16 |  |  | 620 | -0.260 | -0.3942 | No |
| 35 | DAG1 |  |  | 622 | -0.260 | -0.3901 | No |
| 36 | CHMP2A |  |  | 631 | -0.262 | -0.3938 | No |
| 37 | SHROOM3 |  |  | 639 | -0.264 | -0.3964 | No |
| 38 | GNG12 |  |  | 640 | -0.265 | -0.3911 | No |
| 39 | MAGI1 |  |  | 642 | -0.266 | -0.3869 | No |
| 40 | SIPA1L1 |  |  | 645 | -0.267 | -0.3838 | No |
| 41 | NOTCH1 |  |  | 666 | -0.280 | -0.4008 | Yes |
| 42 | PEA15 |  |  | 668 | -0.281 | -0.3962 | Yes |
| 43 | SPRY2 |  |  | 669 | -0.282 | -0.3906 | Yes |
| 44 | CKB |  |  | 676 | -0.287 | -0.3916 | Yes |
| 45 | SEMA6A |  |  | 679 | -0.292 | -0.3880 | Yes |
| 46 | RHOJ |  |  | 682 | -0.293 | -0.3844 | Yes |
| 47 | DDR1 |  |  | 683 | -0.294 | -0.3784 | Yes |
| 48 | GPC4 |  |  | 687 | -0.295 | -0.3759 | Yes |
| 49 | RPS27L |  |  | 688 | -0.295 | -0.3699 | Yes |
| 50 | AKAP12 |  |  | 690 | -0.296 | -0.3651 | Yes |
| 51 | ZFHX4 |  |  | 700 | -0.301 | -0.3692 | Yes |
| 52 | ARAP2 |  |  | 719 | -0.315 | -0.3833 | Yes |
| 53 | BICD1 |  |  | 723 | -0.319 | -0.3803 | Yes |
| 54 | C1orf61 |  |  | 733 | -0.328 | -0.3839 | Yes |
| 55 | HES1 |  |  | 736 | -0.331 | -0.3795 | Yes |
| 56 | PON2 |  |  | 743 | -0.338 | -0.3794 | Yes |
| 57 | MYO6 |  |  | 750 | -0.343 | -0.3793 | Yes |
| 58 | MEIS2 |  |  | 754 | -0.345 | -0.3758 | Yes |
| 59 | SRGAP3 |  |  | 755 | -0.345 | -0.3688 | Yes |
| 60 | FZD3 |  |  | 756 | -0.345 | -0.3619 | Yes |
| 61 | BOC |  |  | 766 | -0.359 | -0.3648 | Yes |
| 62 | MSI2 |  |  | 771 | -0.361 | -0.3621 | Yes |
| 63 | ITGB8 |  |  | 774 | -0.363 | -0.3570 | Yes |
| 64 | MLC1 |  |  | 783 | -0.369 | -0.3587 | Yes |
| 65 | MSMO1 |  |  | 803 | -0.386 | -0.3724 | Yes |
| 66 | MRC2 |  |  | 813 | -0.395 | -0.3746 | Yes |
| 67 | LTBP1 |  |  | 815 | -0.397 | -0.3678 | Yes |
| 68 | HOPX |  |  | 821 | -0.403 | -0.3653 | Yes |
| 69 | DDAH1 |  |  | 828 | -0.413 | -0.3638 | Yes |
| 70 | TTYH3 |  |  | 834 | -0.428 | -0.3608 | Yes |
| 71 | SDCBP |  |  | 845 | -0.438 | -0.3633 | Yes |
| 72 | ZFP36L2 |  |  | 848 | -0.443 | -0.3567 | Yes |
| 73 | TMEM47 |  |  | 855 | -0.447 | -0.3545 | Yes |
| 74 | APC |  |  | 856 | -0.447 | -0.3455 | Yes |
| 75 | SLC1A2 |  |  | 860 | -0.450 | -0.3398 | Yes |
| 76 | DOK5 |  |  | 862 | -0.457 | -0.3317 | Yes |
| 77 | EZR |  |  | 865 | -0.464 | -0.3246 | Yes |
| 78 | SLC35F1 |  |  | 871 | -0.482 | -0.3206 | Yes |
| 79 | ANXA2 |  |  | 872 | -0.482 | -0.3109 | Yes |
| 80 | MOXD1 |  |  | 878 | -0.498 | -0.3065 | Yes |
| 81 | PDGFC |  |  | 883 | -0.515 | -0.3007 | Yes |
| 82 | JUN |  |  | 886 | -0.525 | -0.2924 | Yes |
| 83 | F3 |  |  | 888 | -0.526 | -0.2829 | Yes |
| 84 | CALD1 |  |  | 889 | -0.527 | -0.2723 | Yes |
| 85 | PDPN |  |  | 890 | -0.529 | -0.2617 | Yes |
| 86 | GPM6B |  |  | 897 | -0.542 | -0.2575 | Yes |
| 87 | ATP1B2 |  |  | 904 | -0.558 | -0.2531 | Yes |
| 88 | SAT1 |  |  | 909 | -0.573 | -0.2461 | Yes |
| 89 | DKK3 |  |  | 912 | -0.579 | -0.2367 | Yes |
| 90 | RAB31 |  |  | 916 | -0.586 | -0.2283 | Yes |
| 91 | TTYH1 |  |  | 932 | -0.652 | -0.2321 | Yes |
| 92 | CST3 |  |  | 933 | -0.680 | -0.2184 | Yes |
| 93 | EFNB2 |  |  | 936 | -0.682 | -0.2070 | Yes |
| 94 | CLU |  |  | 938 | -0.685 | -0.1943 | Yes |
| 95 | LRRC17 |  |  | 949 | -0.769 | -0.1901 | Yes |
| 96 | IFITM3 |  |  | 954 | -0.830 | -0.1780 | Yes |
| 97 | CNN3 |  |  | 961 | -0.883 | -0.1670 | Yes |
| 98 | PPP1CB |  |  | 962 | -0.885 | -0.1491 | Yes |
| 99 | TAGLN2 |  |  | 963 | -0.891 | -0.1312 | Yes |
| 100 | ZBTB20 |  |  | 964 | -0.895 | -0.1132 | Yes |
| 101 | LGALS3 |  |  | 970 | -0.989 | -0.0989 | Yes |
| 102 | FABP7 |  |  | 971 | -0.994 | -0.0789 | Yes |
| 103 | SLC1A3 |  |  | 973 | -1.097 | -0.0579 | Yes |
| 104 | CYR61 |  |  | 978 | -1.296 | -0.0364 | Yes |
| 105 | GFAP |  |  | 987 | -2.255 | 0.0000 | Yes |
Table: GSEA details [plain text format]

  

Fig 2: RICHARDS\_NOWAKOWSKI\_SCIENCE\_2017\_VRG\_UPREG: Random ES distribution      
 Gene set null distribution of ES for **RICHARDS\_NOWAKOWSKI\_SCIENCE\_2017\_VRG\_UPREG**
